# On the Use of Local Assessments for Monitoring Centrally Reviewed Endpoints with Missing Data in Clinical Trials^*^

**DOI:** 10.4236/ojs.2013.34A005

**Published:** 2013-08

**Authors:** Sean S. Brummel, Daniel L. Gillen

**Affiliations:** 1 Harvard School of Public Health, Center for Biostatistics in AIDS Research, Boston, USA; 2 Department of Statistics, University of California, Irvine, USA

**Keywords:** Group Sequential, Information, Independent Review, Endpoint, Missing Data

## Abstract

Due to ethical and logistical concerns it is common for data monitoring committees to periodically monitor accruing clinical trial data to assess the safety, and possibly efficacy, of a new experimental treatment. When formalized, monitoring is typically implemented using group sequential methods. In some cases regulatory agencies have required that primary trial analyses should be based solely on the judgment of an independent review committee (IRC). The IRC assessments can produce difficulties for trial monitoring given the time lag typically associated with receiving assessments from the IRC. This results in a missing data problem wherein a surrogate measure of response may provide useful information for interim decisions and future monitoring strategies. In this paper, we present statistical tools that are helpful for monitoring a group sequential clinical trial with missing IRC data. We illustrate the proposed methodology in the case of binary endpoints under various missingness mechanisms including missing completely at random assessments and when missingness depends on the IRC’s measurement.

## 1. Introduction

**Figure 1:**
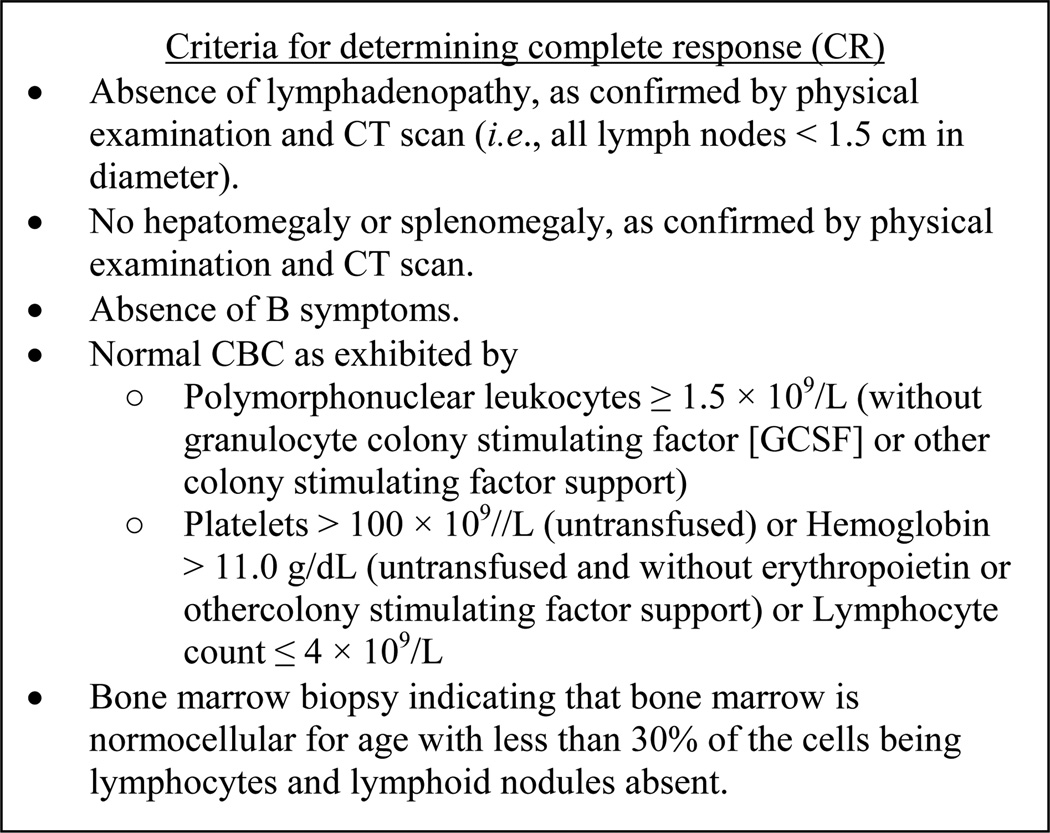
Required criteria for determining a complete response (CR) in chronic lymphocytic leukemia (CLL).

When conducting a clinical trial that utilizes a subclinical and/or subjective primary endpoint it may be necessary to verify the local investigator assignment of the outcome variable. Sometimes this verification is mandated by a regulatory agency or it may be preferred by a study sponsor. The advantage to verify the outcome is that it may decrease misclassification of the outcome in studies performed at multiple sites. As a recent example, consider a phase II clinical trial to investigate the efficacy of an experimental monoclonal antibody in combination with chemotherapy in patients with relapsed chronic lymphocytic leukemia (CLL). A common endpoint in trials targeting CLL is a binary indicator of complete response (CR) of disease following the completion of the therapeutic regime. To standardize the assessment of CR in CLL trials, most studies now use the NCI revised guidelines for determining CR [1], as shown in Figure 1. It is clear that the CR criteria in Figure 1 are subclinical and subjective in nature, requiring radiographic assessment of lymph node size. In this case, the trial’s primary endpoint may be validated by an independent review committee (IRC). A recent a paper by Dodd *et al.* [2] reports an additional seven trials that used an IRC to review the cancer progression measurements of a local investigator: two renal cell carcinoma studies, one colorectal cancer study, and four breast cancer studies.

In the setting of the CLL trial described above, it would not be unusual for an independent data monitoring committee (IDMC) to periodically assess the futility, and possible efficacy, of the experimental intervention through formal hypothesis testing. In this case a group sequential framework would be natural for maintaining frequentist error rates after conducting multiple interim analyses of accruing data. A great deal of research has been conducted in the area of group sequential methods and it is well known that the operating characteristics of a group sequential design depend on, among other things, the exact timing of interim analyses. The timing of sequential analyses is measured by the proportion of statistical information obtained at an interim analysis relative to the maximal information that is anticipated at the final analysis of the trial [3]. Thus it is important to reliably estimate statistical information at each interim analysis in order to properly implement and potentially re-power a chosen group sequential design [4,5]. However, when an IRC is used to adjudicate a trial endpoint there may be a subset of individuals who do not have verified IRC measurements at the time of an interim analysis because the final assessment of their outcome has yet to be returned by the IRC. This results in a portion of trial patients whose primary response from the IRC is missing but whose assessment from the local site (which is typically much quicker to obtain) is known. Relying solely upon validated responses at the time of an interim analysis can result in misleading estimates of statistical information (at best) and opens the possibility of biased estimates of treatment effect (at worst) [2,6]. While the local investigator measurements only serve as a surrogate for the IRC outcome measurements, use of this information on observations that are missing validated outcomes may be helpful in estimating statistical information for sample-size recalculations (also known as sample size reestimation) and for timing future analyses.

In the current manuscript we consider the use of information from local assessments when monitoring an IRC validated binary endpoint such as that encountered in the CLL trial described above. This setting allows us to assess the proposed utility of local assessments in estimating statistical information in clinical trials where a mean-variance relationship exists, and serves as a case study for the importance of information estimation when monitoring a clinical trial with group sequential stopping boundaries. In Section 2 we discuss the importance of accurately estimating statistical information when implementing group sequential stopping rules. This section concludes with an example to illustrate the impact that missing IRC data can have on the operating characteristics of a group sequential design. In Section 3, we propose missing data techniques to aid in estimating statistical information and show how these methods can be used for implementing group sequential tests. In Section 4 we present a simulation study to illustrate the utility of the proposed approach and conclude with a discussion of the challenges of monitoring group sequential clinical trials with IRC validated endpoints.

## 2. The Role of Statistical Information in Implementing Group Sequential Trial Designs

Consider the CLL trial where interest lies in estimating the effect of intervention on the probability of CR (a binary endpoint). Further, suppose that the ratio of the odds of CR comparing intervention to control is used to assess efficacy. Let *Y_ki_* denote the response of individual *i* in treatment arm *k* (*k* = 1 for control, *k* = 2 for intervention) with associated response probabilities given by *p_k_* = Pr[*Y_ki_* = 1]. The odds of CR for group *k* is then given by *Odds_k_* = *p_k_*/(1−*p_k_*), *k* = 1, 2, and the log-odds ratio is given by ψ ≡ log (*Odds*_2_/*Odds*_1_). Finally, suppose that the null hypothesis to be tested is *H*_0_:ψ = 0 against the one-sided alternative *H_a_*:ψ < 0.

Now consider a group sequential test of the above hypothesis. For testing a one-sided alternative, many commonly used group sequential stopping rules consider continuation sets of the form *C_j_* = (*a_j_*, *b_j_*] such that −∞ ≤*a_j_* ≤*b_j_* ≤ ∞ for *j* = 1,⋯, *J* analyses. These boundaries may be interpreted as the critical values for a decision rule. For instance, in the CLL trial a test statistic less than *a_j_* would correspond to a decision in favor of superiority of the intervention while a test statistic exceeding *b_j_* would correspond to a decision of futility regarding the intervention. Particular families of group sequential designs correspond to parameterized boundary functions that relate the stopping boundaries at successive analyses according to the proportion of statistical information accrued. For instance, in the context of the CLL trial, if we calculate a normalized statistic $Z_{j}={\mathrm{\psi ̂}}_{j}/\sqrt{\text{Var}[{\mathrm{\psi ̂}}_{j}]}$ where ψ̂_*j*_ is the maximum likelihood estimate of the log-odds ratio computed at analysis *j* with corresponding variance Var[ψ̂_*j*_], the proportion of statistical information accrued at analysis *j* can be calculated as П_*j*_ ≡ Var[ψ̂_*J*_]/Var[ψ̂_*j*_] where Var[ψ̂_*j*_] is the variance of the maximum likelihood estimate of the log-odds ratio computed at the final analysis of the trial under a presumed maximal sample size. That is, П_*j*_ represents the fraction of total statistical information, defined as the inverse of the variance of the final odds ratio estimate, available from all patients at the time of interim analysis *j*. It then follows that for some specified parametric functions f_*_ (·), the critical values for a decision rule at analysis *j* can be given by *a_j_* = *f_a_* (П_*j*_) and *b_j_* = *f_b_* (П_*j*_). For critical values on the normalized Z-statistic scale, popular examples of f_*_ (·) include a one-sided version of the Pocock [7] stopping rule that takes *f_a_* (П_*j*_) = −*G*, *f_b_* (П_*j*_) = ∞ and a one-sided version of the O’Brien-Fleming [8] stopping rule that takes $f_{a}(\prod_{j})=-G\prod_{j}^{-1/2},f_{b}(\prod_{j})=\infty$, where in both cases the value of *G* is chosen to maintain a pre-specified type I error rate.

The choice of a stopping rule is generally based upon the assessment of a wide range of statistical operating characteristics across multiple candidate designs [3]. In addition to type I error, commonly considered frequentist operating characteristics include power, stopping probabilities at each analysis, and average sample size. These characteristics depend on the sampling distribution of the test statistic under a given group sequential sampling design. Unlike a fixed sample design where a single hypothesis test is performed after the accrual of all trial data, the sampling density of a test statistic in a group sequential framework not only depends upon the total amount of statistical information accrued over the entire trial but also on the timing of interim analyses as measured by the proportion of the trial’s maximal statistical information, П_*j*_, attained at each interim analysis [3]. Because of this, there are usually at least two complicating factors that must be dealt with during the monitoring of a clinical trial. First, the schedule of interim analyses may not follow the schedule assumed during the design of the trial. Often, meetings of an IDMC are scheduled according to calendar time, and thus the sample size available for analysis at any given meeting is a random variable. Similarly, accrual may be slower or faster than planned, thereby resulting in a different number of interim analyses than was originally planned. Because the exact stopping boundaries are dependent upon the number and timing of analyses, either of these scenarios will necessitate modifications of the stopping rule. Second, the estimate for response variability that was assumed at the design phase is typically incorrect. As the trial progresses, more accurate estimates may be obtained using available data at each interim analysis. In this case, if one wishes to maintain the originally specified power of the trial then updates to the maximal sample size may be necessary due to changes in variance estimates. Of course, changes in maximal sample size will result in changes to the proportion of information at all previously conducted analyses.

Two ways to adjust for deviations in the timing of planned analyses in order to maintain some of the trial’s original operating characteristics include the error spending approach [9] and the constrained boundaries algorithm [5]. First and foremost, these methods are primarily used to maintain the size of the trial (type I error). A choice must then be made as to whether the maximal sample size or the power to detect a clinically relevant alternative should be maintained. Briefly, the constrained boundaries algorithm for maintaining the power of a one-sided group sequential hypothesis test is implemented as follows: At the design stage, boundary shape functions are specified as *f_a_* (П_*j*_) and *f_b_* (П_*j*_), where П_*j*_ denotes the planned proportion of maximal statistical information attained at interim analysis *j*, *j* = 1,⋯, *J* with П_*J*_ = 1. At the first analysis П_1_ is determined, and stopping boundaries *a*_1_ and *b*_1_ are computed. A schedule of future analyses, П_2_,⋯, П_*J*_, which may differ from the originally assumed schedule of analyses is then assumed and a stopping rule using the design parametric family f_*_ (·) (constraining the first boundaries to be *a*_1_ and *b*_1_) is found which has the desired power. This consists of searching for a new maximal sample size that has the correct type I error and power to detect the alternative for the parametric design family for the assumed schedule of interim analyses. At later analyses, the exact stopping boundaries used at previously conducted interim analyses are used as exact constraints at those analysis times, and the stopping boundaries at the current and all future analyses as well as the new maximal sample size needed to maintain statistical power are re-computed using the parametric family of designs specified at the design stage and an assumed schedule of future analysis times. Reference [5] notes that when *f_a_* (П_*j*_) and *f_b_* (П_*j*_) are defined on the type I and II error spending scales, this procedure is equivalent to the error spending approach given in reference [10].

As noted above, in cases where power is to be maintained the current best estimate of the variance of the response variable at each interim analysis is typically used in place of the variance assumed at the design stage. Use of a more accurate estimate of the response variability, and hence statistical information, at earlier analyses provides more accurate estimates of the maximal sample size, *N_J_*, at earlier analyses. This will in turn lead to less variation in the relative timing of analyses as the trial proceeds and *N_J_* is updated. In the context of the motivating CLL trial the variability associated with a single sampling unit's response is dependent upon the unit’s IRC response probability. Specifically, if *Y_ki_* denotes the response of individual *i* in treatment arm *k* (*k* = 1 for control, *k* = 2 for antibody) then Var[*Y_ki_*] = *p_k_* (1 −*p_k_*), where *p_k_* is the response probability for group *k*. The result is that biased estimates of response probabilities at an interim analysis will lead to biased estimates of the variability associated with the response variable. To see the implication of this, consider the case where the constrained boundaries algorithm described above is used to maintain statistical power by updating the trial’s maximal sample size using a biased estimate of response variability and statistical information. At the time of an interim analysis, missing IRC validated outcomes may be more or less likely to be positive when compared to observed IRC outcomes. This may occur because positive outcomes often require an additional radiologic reading for confirmation, thus leading to a lagged reporting time. In this case, using only data on the available IRC outcomes would lead to downward bias in the event rate, and hence bias in the estimate of statistical information. The end result may be a tremendously (under-) overpowered study depending on the magnitude and direction of the bias.

## 3. Example of the Impact of Missing Data

In this section we demonstrate the impact on group design operating characteristics when the timing of implemented interim analyses deviates from the originally planned analysis schedule. Using parameters similar to those that we have encountered in a previously conducted CLL trial, we consider a level 0.05 test of the null hypothesis *H*_0_:ψ = 0 against a lesser alternative *H_a_*:ψ < 0, where ψ denotes the log-odds ratio comparing intervention to control. We consider a study design with 95% power for detecting a true odds ratio of 0.65 (ψ = −0.43) under an assumed event rate of 0.2 in the control arm. We further consider implementing 4 analyses that are equally spaced in information time. That is, the desired analysis schedule at the design phase is specified by П = {0.25, 0.5, 0.75, 1}.

**Figure 2:**
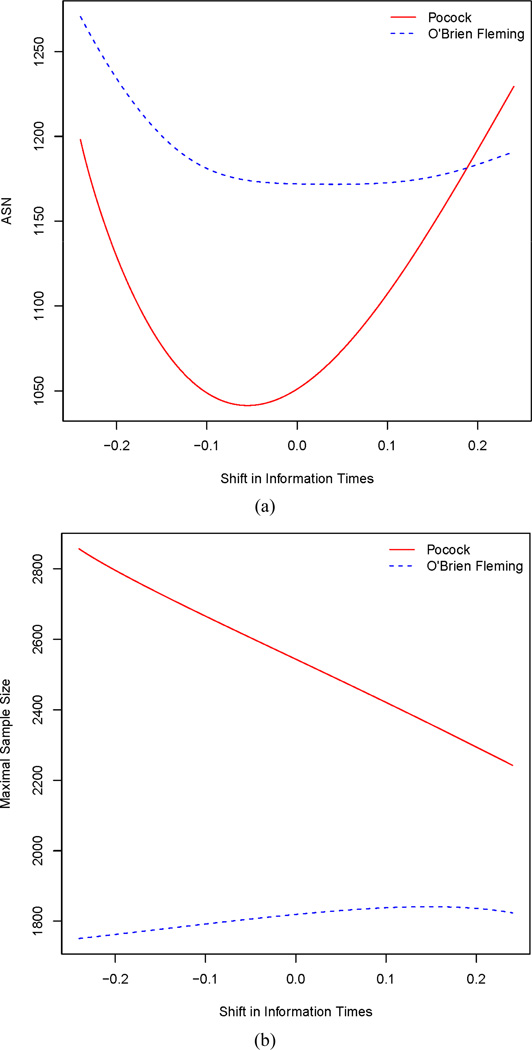
Effects of shifting information time for the first three of four analyses on information time on ASN and maximal sample size evaluated under the alternative hypothesis ψ = −0.43. The x-axis is the *l* value in Π = {0.25 + *l*, 0.5 + *l*, 0.75 + *l*, 1}. (a) Effect on ASN; (b) Effect on maximal sample size.

To illustrate the impact of changing the timing of analyses we consider a shift parameter *l* so that П = {0.25 + *l*, 0.5 + *l*, 0.75 + *l*, 1}. Under the alternative hypothesis, Figure 2 depicts the maximal sample size and the average sample number (ASN) for the symmetric O'Brien-Fleming and Pocock designs as the timing of analyses shifts away from the originally desired equally spaced setting (*l* = 0). Figure 2(a) shows that the minimum ASN attained by the O’Brien-Fleming design occurs at values of *l* between −0.1 and 0.1, while the minimum ASN for the Pocock design occurs at approximately *l* = −0.06. In addition, Figure 2(b) shows that the maximal sample size for the O’Brien-Fleming design is fairly robust to the timing of analyses. It is clear that the ASN and maximal sample size for the Pocock design is more sensitive to shifts in the analysis timing when compared to the O’Brien-Fleming design. This is because the Pocock is far less conservative at early analyses when compared to the O’Brien-Fleming design.

From Figure 2 it is clear that changes in the timing of analyses will affect the operating characteristics of a statistical design.

We now consider a single simulated example to demonstrate the implementation of the constrained boundaries approach for trial monitoring and how the stopping boundaries of a planned design and an implemented design can differ due to the estimation of information at interim analyses when this approach is utilized. For this example, a shift in the total information schedule from analysis to analysis will be due to an underestimation of a success probability for a binary endpoint, resulting from missing data. When monitoring a clinical trial with an IRC adjudicated endpoint missing data is likely due to lagged IRC response data. As such, IRC outcomes would be more frequently missing at early analyses, with complete data at the final analysis. In this case higher bias in the estimated probabilities would be seen at earlier analyses. For illustration purposes the example assumes that only those who would have been classified as having an event by the IRC will have the possibility of being missing. The result is that the event probabilities will be underestimated at each analysis and these estimates will trend upward from analysis to analysis until the final analysis where complete data will be available on all subjects. Specifically we assume that 39%, 16%, and 3% of IRC endpoints are missing at the first, second, and third interim analyses; and no IRC endpoints are missing at the final analysis. This setting reflects a similar scenario to trials we have previously monitored.

We focus on a symmetric O’Brien-Fleming stopping rule with 4 equally spaced analyses, allowing early stopping for efficacy and futility, and 95% power for detecting an odds ratio of 0.65. This design specification results in a maximal sample size of 1819 patients. In monitoring the trial we consider re-powering the study at each interim analysis using the constrained boundaries approach of [5] as described in Section 2. For this example, at the first interim analysis the estimated event rates are ${\mathrm{p̂}}_{1}^{\left(1\right)}=0.110$ and ${\mathrm{p̂}}_{2}^{\left(1\right)}=0.096$ with a sample size of 436. With these observed estimates the study is then re-powered with a new maximal sample size of 2705 in order to maintain 95% power for detecting an odds ratio of 0.65. This results in a smaller proportion of information at the first analysis than originally planned (25% to 16%). Using this estimate of information along with the current best estimate of variability, the efficacy and futility boundaries at the first interim analysis are recomputed to be 0.26 and 2.47, respectively, under the pre-specified symmetric O’Brien-Fleming parametric stopping rule. The observed odds ratio at the first analysis, ${\hat{OR}}_{1}$, is 0.86 and this value lies within the continuation region of the stopping rule. At the second analysis, with data now available on 1145 subjects, the observed success probabilities are ${\mathrm{p̂}}_{1}^{\left(2\right)}=0.146$ and ${\mathrm{p̂}}_{2}^{\left(2\right)}=0.122$. These probabilities are higher than those observed at the first analysis, resulting in a reduction in the re-computed maximal sample size needed to maintain 95% power for detecting an odds ratio of 0.65. The newly re-computed maximum sample size is reduced to 2176, and the percentage of information for the first two interim analyses shifts to 20% for the first analysis and 53% for the second. Constraining on the first decision boundaries (shown in Table 1), the efficacy and futility boundaries at the second analysis are now computed to be 0.66 and 0.98, respectively. The observed odds ratio at this analysis is 0.81, again implying continuation of the trial. As before, the study is re-powered at the third analysis and then continues to the final analysis where the final sample size is ultimately 1945 subjects. The final sample size is larger than what was assumed at the design stage due to the shifts in the timing of analyses that resulted from underestimation of the response probabilities at early analyses.

**Figure 3:**
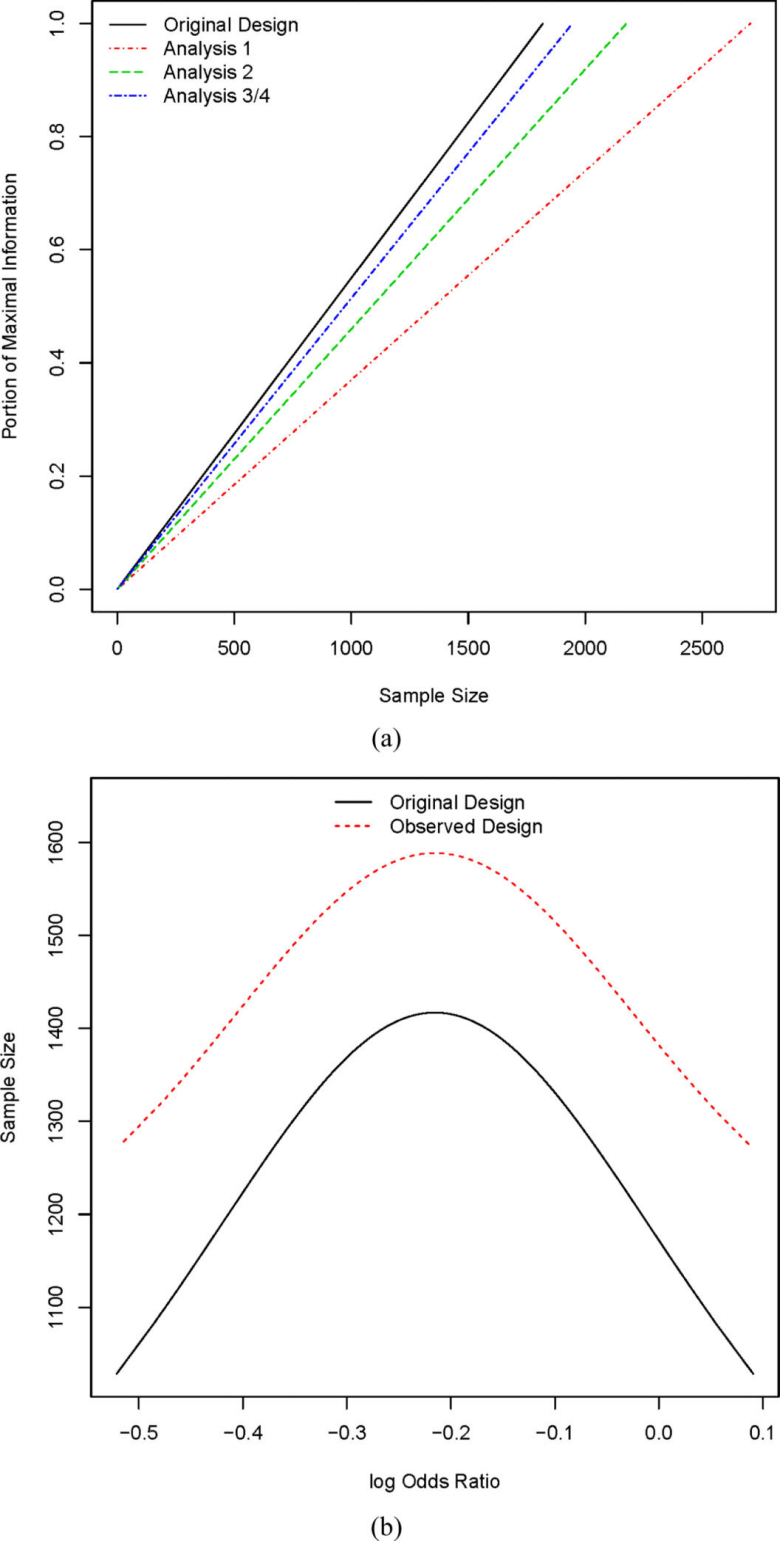
(a) Estimates of information growth at each analysis. Differences are due to changes estimates of event rates and recalculating maximal sample size. (b) Deviations in ASN due to changes in the proportion of maximal information as a function of the log-odds.

Had unbiased estimates of the success probabilities at early analyses been available, the total sample size for the trial presented in Table 1 would have been much closer to that of the original design specification. Figure 3 shows the estimated information growth curve at each analysis for the trial. As can be seen in this plot, at the first analysis, the information growth has substantially changed from the planned portioning of information. The change in information growth is due to a recalculated maximal sample size, but this recalculation was only necessary because of the underestimated probabilities of success. Specifically, the recalculated maximal sample size at analysis one is much larger than the maximal sample size from the original design. This change in the maximal sample size is due to the dependence of the vaiance of the log-odds ratio on the underlying probabilities of success. However, at the third analysis, the information growth is approximately equal to the original design. Ultimately, both the original and observed design have similar maximal sample sizes, but the ASN, as seen in Figure 3 differs substantially. Specifically, the changes in ASN are due to the observed design not following the original intent of having four analyses that are spaced evenly with respect to information time. In turn, the changes in information alter the decision boundaries, as previously discussed. Ultimately, trials with different boundaries and information levels will have different probabilities of stopping at a given analysis, resulting in different operating characteristics.

## 4. Using Local Investigator Assessment to Monitor Study Data with Missing IRC Assessments

Had unbiased estimates of the underlying success probabilities been available at early analyses in the previous example the resulting changes to the maximal sample size would have been unnecessary. This would have resulted in decision boundaries similar to those originally specified at the design stage. In this section we discuss methods to improve the estimation of information using all of the observed local investigator assessments.

When monitoring an IRC-validated primary endpoint, a reasonable approach might perform hypothesis testing using only complete IRC measurements but would use a missing data model that incorporates local investigator assessments in order to estimate response probabilities and hence statistical information. Provided that local assessment is predictive of the IRC-validated outcome, incorporation of local investigator assessment into the estimation of statistical information will result in improved estimates of statistical information, potentially minimizing changes to the trial design’s original operating characteristics. Further, by only using the investigator assessment testing is based solely on observed IRC-validated data.

For ease of exposition we consider the use of local investigator assessments when an IRC response is missing at a specific interim analysis and drop the analysis subscript. Assume that at a given interim analysis local investigator assessments are available for *n_k_* subjects in group *k*, and without loss of generality assume that complete data are available for the first *r_k_* subjects while the remaining *n_k_* − *r_k_* subjects are missing an IRC assessment, *k* = 1,2. For complete pairs let ***y**_ki_* = (*y*_*ki*1_, *y*_*ki*2_) denote the vector of binary local response (*y*_*ki*1_) and binary IRC response (*y*_*ki*2_) for subject *i* in group *k*, *i* = 1, ⋯, *r_k_*, *k* = 1,2. For subjects with only a local assessment and no IRC response, let ***z**_ki_* = (*z*_*ki*1_, *y*_*ki*2_), where *z*_*ki*1_ is the unobserved IRC response, *i* = *r_k_* + 1, ⋯, *n_k_*, *k* = 1,2. The total data available for group *k* can then be summarized in the contingency tables provided in Table 2, where for complete cases

$$n_{k00}=\sum_{i=1}^{r_{k}}(1-y_{ki1})(1-y_{ki2}),n_{k10}=\sum_{i=1}^{r_{k}}y_{ki1}(1-y_{ki2}),\;n_{k01}=\sum_{i=1}^{r_{k}}(1-y_{ki1})y_{ki2},\text{and }n_{k11}=\sum_{i=1}^{r_{k}}y_{ki1}y_{ki2}.$$

The unobserved cell counts for the incomplete cases are defined analogously as

$$M_{k00}=\sum_{i=r_{k}+1}^{n_{k}}(1-z_{ki1})(1-y_{ki2}),M_{k10}=\sum_{i=r_{k}+1}^{n_{k}}z_{ki1}(1-y_{ki2}),\;M_{k01}=\sum_{i=r_{k}+1}^{n_{k}}(1-z_{ki1})y_{ki2},\text{and }M_{k11}=\sum_{i=r_{k}+1}^{n_{k}}z_{ki1}y_{ki2}.$$

In the context of the current problem, the common success probabilities

$$p_{kab}=\text{Pr}[Y_{ki1}=a\text{ and }Y_{ki2}=b]=\text{Pr}[Z_{ki1}=a\text{ and }Y_{ki2}=b],a,b=0,1,$$

must be estimated for study monitoring. Of course we do not observe *M_kab_*. However, since the local assessments are observed, the marginal totals *m*_*k*·0_ and *m*_*k*·1_ are known, and conditional on *n_kab_* and *m*_*k·b*_, $M_{kab}|y,z\sim \text{Bin}(p_{kab}^{*}\equiv p_{kab}/p_{k\cdot b},m_{k\cdot b})$, *a,b* = 0,1.

For the remainder of this section we consider three of many possible procedures to estimate ***p**_k_* = (*p*_*k*00_, *p*_*k*01_, *p*_*k*10_, *p*_*k*11_) when missing IRC data are present at an interim analysis. Once estimated, ***p̂**_k_* can then be used to estimate the sampling variability of a response and hence the available statistical information for sample size adjustment and planning of future analyses.

### 4.1. Expectation Maximization Algorithm (EM)

The EM algorithm [11] is a well-known approach for finding maximum likelihood estimates in the presence of missing data. Briefly, the EM algorithm augments the observed data likelihood with missing data so that maximum likelihood estimates are easily found. That is, we assume an augmented likelihood *L*(***p**_k_*|*Y, Z*). We then compute the expected value (E-step) of the log-augmented likelihood with respect to the missing IRC data, conditional on the observed data and the current iteration value for ***p**_k_*. In the M-Step, the log-augmented likelihood is maximized as if the conditional expectations were observed data. The E- and M-steps are repeated until convergence to get our estimate ***p̂**_k_* for ***p**_k_*.

Symbolically, for an initial estimate for ***p**_k_*, $p_{k}^{l}$, the estimate of ***p**_k_* is updated using the following algorithm,

$$E-\text{Step}:Q(p_{k},p_{k}^{l})=E_{Z|Y,p_{k}^{l}}[\text{log}\{L(p_{k}|Y,Z)\}]\;M-\text{Step}:p_{k}^{l+1}={\text{argmax}}_{p_{k}}Q(p_{k},p_{k}^{l}),$$

where *Y* and *Z* denote all observed and unobserved data on local and IRC responses. The algorithm is repeated until a distance metric between $p_{k}^{l+1}$ and $p_{k}^{l}$ is small, and the final estimate for ***p**_k_* is given by ${\mathrm{p̂}}_{k}=p_{k}^{l+1}$. Appendix 7.1 provides more detailed steps of the EM algorithm to maximize a multinomial likelihood to obtain estimates ***p**_k_* of when there are missing IRC data.

### 4.2. Multiple Imputation

Multiple imputation is another natural approach to account for missing data. To perform multiple imputation in the case of missing IRC assessments we can first model the conditional distribution *Z*_*ki*1_|*Y, **p**_k_* and impute the missing data from this distribution *D* times to obtain *D* estimates of ***p**_k_*. In this manuscript we find the conditional distribution by using regression estimates from regressing the IRC data on the local investigator data. The estimator for ***p**_k_* is calculated from ${\bar{p}}_{k}=\frac{1}{D}\sum_{d=1}^{D}{\mathrm{p̂}}_{k}^{\left(d\right)}$, where ${\mathrm{p̂}}_{k}^{\left(d\right)}$ is the *d^th^* imputation estimate of ***p**_k_*.

Multiple imputation can be carried out in the multinomial example above by imputing the missing *z_kij_* values using a binomial distribution. One possibility is to use logistic regression for the imputation model. In this case we fit a logistic model using the complete data with the IRC data as the outcome and the local investigator data as a predictor. Letting α̂_*k*_ and β̂_*k*_ denote the estimated intercept and slope of the fitted logistic regression model for group *k*, the missing data can be imputed at the individual level as

$$Z_{ki1}|Y=y_{ki2}\sim \text{Bernoulli}(\frac{e^{{\mathrm{\alpha ̂}}_{k}+{\mathrm{\beta ̂}}_{k}y_{ki2}}}{1+e^{{\mathrm{\alpha ̂}}_{k}+{\mathrm{\beta ̂}}_{k}y_{ki2}}}).$$

### 4.3. Complete Case Analysis

The last method that we consider is the complete case analysis. This method is the simplest, as it only analyzes the complete data. While this method represents current practice, it assumes that missingness is missing completely at random (MCAR, [12]) and ignores potentially useful information in local response data. In this case, *p̂_kab_* is simply given by *n_kab_*/*r_k_*.

## 5. Simulation Study

In Section 3 we demonstrated that the operating characteristics of a group sequential design depend on the timing of interim analyses and showed how changes from planned information can occur when estimates of response probabilities are biased. In this section we present a simulation study to illustrate the type one error rate, power, ASN, and 75th percentile of the sample size distribution using the three approaches for incorporating local investigator assessments that were described in Section 4.

Following from the previous sections, focus is on testing the IRC validated log-odds ratio comparing control to antibody in the context of the CLL trial. Specifically, we consider testing a one sided lower alternative with a type one error rate of 5% and 95% power for the design alternative of −0.43. The stopping rule is taken to be a symmetric O’Brien-Fleming design with four equally spaced interim analyses that allow for early stopping in favor of futility or efficacy. The simulations are set so that the true odds ratio is 0.65, comparing antibody to control, regardless of whether outcomes are based on the local investigator or the IRC. However, the control arm event rate was assumed to be 0.20 for the IRC and 0.25 for the Local investigator. The missing data was defined differently to illustrate three missing data mechanisms: MCAR, missing at random (MAR), and not missing at random (NMAR). Under MAR the probability of missing an IRC outcome depends on the assigned event assessment of the local investigator. Under NMAR only positive IRC outcomes have the potential to be missing. In the MCAR simulation, at the first analysis, the probability of a missing IRC response was taken to be 17.5%. In the MAR simulations, at the first analysis, the probability that a positive IRC response was missing was taken to be 35% if the local investigator response was positive. Lastly, under NMAR, at the first analysis, the probability that a positive IRC response was missing was taken to be 35%, regardless of the local investigator response. Since interim tests are on accumulating data, the proportion of missing responses decreases with each analysis as all of the patients reach the time for evaluation.

For the log-odds ratio, in contrast to the binomial variance, the variance of the estimator increases as the success probability moves away from 0.5. Given that the probability of a response was taken to be less than 0.5 in the simulation study, and because observed IRC response rates are biased downwards under the MAR and NMAR setups, the variance of the odds ratio will decrease as the trial continues. Thus a re-powering of the trial will result in an increase in the maximal sample size. However, because an unbounded maximal sample size is unrealistic in practice (a study sponsor is sure to have logistical and financial constraints), the maximal sample size was constrained so that it would not be larger than 1.25 times the originally planned maximal sample size (*N*_max_ = 1812, *ASN_null_* = *ASN_alt_* = 1172). If this restriction is removed, the observed differences between the missing data models would be more extreme.

The simulations reflect a scenario where the investigators are not expecting any missing information at the design stage of the trial. Thus, at the first analysis, all of the scenarios analyze the data at 0.25 × *N*_max_. However, due to missingness less than 25% of the originally planned maximal information is observed at the first analysis. The action is then taken to test the data at the current amount of information then recalculate maximal sample size to maintain power and plan for future analyses. Results are based upon 10,000 for each scenario.

Table 3 depicts the results from the simulation study. Along the rows we consider the three missing data mechanisms (MCAR, MAR and NMAR). Under the column Future Timing in Table 3, we consider two ways to select the next interim analysis sample size: oversampling in anticipation of missing data (“Predict Info”), and ignoring the possibility of future missing data (“Info ∝ *N*”). The later scenario is included to illustrate that the primary advantage of incorporating local investigator assessments is in the sample size computation at the first analysis time.

The three considered monitoring strategies are tabulated along the columns of Table 3. As can be seen, all of the approaches exhibit the desired type one error rates. However, when the data are NMAR the simulations show that the power is higher than the specified 95%, ranging from 96% to 97%.

Next we discuss the efficiency of the EM algorithm and multiple imputation approaches relative to available case analysis. Under the MCAR setting, the sample size statistics are roughly equal across each of the strategies for estimating statistical information (Null: *ASN_Complete_* = 1102, *ASN_MI_* = 1101, *ASN_EM_* = 1102). This is to be expected since the estimates of variability are valid under MCAR for all of the missing data models. In the MAR simulations, the sample size statistics show a larger savings in ASN when local investigator assessments are used to estimate statistical information (Alt: *ASN_Complete_* = 1265, *ASN_MI_* = 1212, *ASN_EM_* = 1211). These differences are due to the fact that the available case statistic tends to overestimate the variability associated with the final test statistic and project future analyses much too far into the future. Similar patterns are observed for the NMAR scenarios. We note that the lower sample size estimates relative to the “Predict Info” scenario is due to an overall shift in the originally proposed analysis times.

## 6. Discussion

It is becoming increasingly common for regulatory agencies to demand independent verification of study response in clinical trials that utilize a subclinical and/or subjective primary endpoint. Attaining IRC validation in these cases can result in significantly lagged data. The result is that during the monitoring of a trial, IRC-validated data may only be available on a subset of patients which local investigator assessment of the primary outcome is known at the time of an interim analysis. A further complicating issue is that the observed IRC lag time may be dependent upon the response. For example, positive responses for disease progression in cancer studies may require an additional radiologic reading. This scenario can result in biased estimates of the overall response probability at the time of interim analysis, resulting in erroneous changes to the study’s maximal sample size if the study is to be repowered. In the current manuscript, we illustrated issues with the use of local investigator assessments to re-estimate maximal sample size at the time of an interim analysis. Specifically, we considered three different methods for dealing with missing data that can arise when an IRC is used to validate local investigator response measurements. We have shown that using local investigator assignment of an outcome variable can be helpful when monitoring a group sequential trial by obtaining more precise estimates of information. When testing is based upon only complete cases and local assessments are used to improve information estimates, the proposed methods do not affect type one error rates, ASN, or power when missing IRC-validated outcomes are MCAR. However, when missing data are MAR or NMAR, use of local investigator assessments to estimate study response rates for the purposes of recomputing maximal sample size can be helpful in maintaining the planned operating characteristics of the design. In addition, since the true information will be known at the final analysis, type one error rates will be robust when using a miss-specified missing data model.

Relative to the complete case analysis, use of local assessments for recomputing maximal sample size resulted in generally lower sample sizes (summarized by ASN and the 75th percentile of the sample size distribution) with little observed change in type I and II error rates. This is a result of lower observed event rates due to the missingness mechanism that was considered. In this case, early analyses that only use complete cases would tend to compute large sample size re-estimates to maintain study power while accounting for the low event rate. This, in turn, pushes future analyses back in information time resulting in generally higher sample sizes. In our experience this is a realistic scenario because missing IRC-validated outcomes tend to have a higher probability of being a positive response since these cases generally require more time and additional radiologic readings.

The methods presented in this manuscript are easily implemented using any group sequential package that implements the constrained boundaries approach of [5]. One example is the **RCTdesign** package for the **R** statistical programming language or **S+SeqTrial**. Example code for computing decision boundaries at the first analysis while updating information using multiple imputation is presented in the Appendix. The **RCTdesign** package is freely available by request from the authors of http://www.rctdesign.org.

We have only advocated using local assessments to predict study response probabilities in order to obtain more precise estimates of statistical information. Another potential strategy when monitoring a test statistic with missing data is to test the imputed statistic; however, such an approach would be controversial for primary hypothesis testing since final inference would then be dependent upon a correctly specified missing data model. Further investigation of the use of local investigator assessments for estimating treatment effect remains area of open research. In addition, priors for the discordance between the local investigator and IRC measurements could be used at the design stage if available to help correct for the issues discussed in this text. This also remains an area of open research.

## Figures and Tables

**Table 1:** Example of planned and implemented stopping boundaries when statistical information is biased due to missing data. The planned design is a one-sided symmetric O’Brien-Fleming design with 95% power for an odds ratio of 0.65. The observed design is the implemented design. Π is the (biased) estimated proportion of information. *p̂*_1_ and *p̂*_2_ denote the probability estimates for the control and antibody arms, respectively.

Analysis (*j*)	1	2	3	4
**Planned Design**				
*p*_1_ = 0.20, *p*_2_ = 0.14, *OR* = 0.65				
Sample Size	454.8	909.61	1364.41	1816.22
Information Fraction (Π_*j*_)	0.25	0.50	0.75	1.00
Decision Boundary Efficacy (Odds-scale)	0.42	0.65	0.075	0.81
Decision Boundary Futility (Odds-scale)	1.54	1.00	0.86	0.81
**Implemented Design**				
*Analysis* 1				
*p̂*_1_ = 0.110, *p̂*_2_ = 0.096, $\hat{OR}=0.86$, *Z* = −0.49				
Sample Size	436	1192	1949	2705
Information Fraction (Π_*j*_)	0.16	0.44	0.72	1.00
Decision Boundary Efficacy (Odds-scale)	0.26	0.61	0.74	0.81
Decision Boundary Futility (Odds-scale)	2.47	1.06	0.88	0.81
*Analysis* 2				
*p̂*_1_ = 0.146, *p̂*_2_ = 0.122, $\hat{OR}=0.81$, *Z* = −1.19				
Sample Size	436	1145	1660	2176
Information Fraction (Π_*j*_)	0.20	0.53	0.76	1.00
Decision Boundary Efficacy (Odds-scale)	0.26	0.66	0.75	0.81
Decision Boundary Futility (Odds-scale)	2.47	0.98	0.86	0.81
*Analysis* 3				
*p̂*_1_ = 0.165, *p̂*_2_ = 0.136, $\hat{OR}=0.80$, *Z* = −1.63				
Sample Size	436	1145	1631	1945
Information Fraction (Π_*j*_)	0.22	0.59	0.84	1.00
Decision Boundary Efficacy (Odds-scale)	0.26	0.66	0.77	0.81
Decision Boundary Futility (Odds-scale)	2.47	0.98	0.84	0.81
*Analysis* 4				
*p̂*_1_ = 0.170, *p̂*_2_ = 0.140, $\hat{OR}=0.79$, *Z* = −1.83				
Sample Size	436	1145	1631	1945
Information Fraction (Π_*j*_)	0.23	0.59	0.84	1.00
Decision Boundary Efficacy (Odds-scale)	0.26	0.66	0.77	0.81
Decision Boundary Futility (Odds-scale)	2.47	0.98	0.84	0.81

**Table 2:** Aggregated complete and incomplete observations for group *k, k* = 1, 2 from a clinical trial with lagged IRC response data.

	Review Type			Local Data	
Complete Cases			No Event	Event	Total

	IRC Data	No Event	*n*_*k*00_	*n*_*k*01_	*n*_*k*0·_
	Data	Event	*n*_*k*10_	*n*_*k*11_	*n*_*k*1·_
		Total	*n*_*k*·0_	*n*_*k*·1_	*r_k_*

Incomplete Cases			No Event	Event	Total

	IRC Data	No Event	*M*_*k*00_	*M*_*k*01_	*M*_*k*0·_
	Data	Event	*M*_*k*10_	*M*_*k*11_	*M*_*k*1·_
		Total	*M*_*k*·0_	*M*_*k*·1_	*n_k_* − *r_k_*

**Table 3:** Simulations under MCAR, MAR, and NMAR showing type one error rates, power, ASN, and the seventy fifth percentile of the sample distribution for the available case analysis, multiple imputation, and the EM algorithm. Results are based on 10,000 simulated trials under each scenario.

			Information Estimation								
			
Simulation	Parameter	Future Timing	Complete Cases			Multiple Imputation			EM		

			Reject	ASN	75% Sample	Reject	ASN	75% Sample	Reject	ASN	75% Sample
MCAR	Null	Predict Info	0.045	1102	1314	0.045	1101	1314	0.045	1102	1314
		Info ∝ *N*	0.050	1075	1208	0.050	1077	1206	0.052	1078	1314
	Alt	Predict Info	0.944	1286	1498	0.945	1285	1496	0.944	1286	1496
		Info ∝ *N*	0.951	1260	1420	0.950	1256	1412	0.951	1261	1420
MAR	Null	Predict Info	0.047	1084	1260	0.047	1055	1274	0.048	1053	1270
		Info ∝ *N*	0.046	1057	1196	0.047	1040	1204	0.048	1038	1202
	Alt	Predict Info	0.959	1265	1450	0.952	1212	1436	0.953	1211	1436
		Info ∝ *N*	0.956	1240	1424	0.951	1188	1366	0.953	1188	1366
NMAR	Null	Predict Info	0.050	1205	1314	0.050	1124	1278	0.051	1125	1280
		Info ∝ *N*	0.048	1182	1274	0.048	1099	1220	0.049	1100	1220
	Alt	Predict Info	0.972	1340	1718	0.963	1290	1534	0.964	1292	1616
		Info ∝ *N*	0.972	1321	1640	0.964	1271	1638	0.963	1274	1638

## Appendix

### A1. Steps for the EM Algorithm

In the context of using local assessments to estimate IRC response probabilities it is straightforward to compute the conditional expectation of *Z* given *Y*. Using the notation of Section 4 and omitting the group indicator, the augmented likelihood is given by, $L(p|Y,Z)=p_{00}^{n_{00}+M_{00}}p_{01}^{n_{01}+M_{01}}p_{10}^{n_{10}+M_{10}}p_{11}^{n_{11}+M_{11}}$, And log-augmented likelihood is then

$$\text{log}[L(p|Y,Z)]=(n_{00}+M_{00})\text{log}(p_{00})+(n_{01}+M_{01})\text{log}(p_{01})+(n_{10}+M_{10})\text{log}(p_{10})+(n_{11}+M_{11})\text{log}(p_{11}).$$

The log-augmented likelihood is linear with respect to *M_ab_*, *a,b* = 0,1, so the expected value is straightforward to compute. Thus, the conditional expectation of the log-likelihood (E-Step) results in

$$Q(p,p^{l})=(n_{00}+m_{\cdot 0}p_{00}^{*})\text{log}(p_{00})+(n_{01}+m_{\cdot 1}p_{01}^{*})\text{log}(p_{01})+(n_{10}+m_{\cdot 0}p_{10}^{*})\text{log}(p_{10})+(n_{11}+m_{\cdot 1}p_{11}^{*})\text{log}(p_{11}),$$

with $p_{ab}^{*}=p_{ab}/p_{\cdot b}$.

For the M-step, maximizing *Q*(*p, p^l^*) yields,

$$p_{ab}^{l+1}=(n_{ab}+m_{\cdot b}p_{ab}^{*,l})/n=(n_{ab}+m_{\cdot j}p_{ab}^{l}/p_{\cdot b}^{l})/n,a,b=0,1.$$
